# Study protocol for a multi-component kindergarten-based intervention to promote healthy diets in toddlers: a cluster randomized trial

**DOI:** 10.1186/s12889-016-2952-x

**Published:** 2016-03-17

**Authors:** Sissel H. Helland, Elling Bere, Nina Cecilie Øverby

**Affiliations:** Department of Public health, Sport and Nutrition, Faculty of Health and Sport Sciences, University of Agder, PO Box 422, 4604 Kristiansand, Norway

**Keywords:** Food neophobia, Diet variety, Toddlers, Parental feeding practices, Kindergarten, Overweight

## Abstract

**Background:**

There is concern about the lack of diversity in children’s diets, particularly low intakes of fruit and vegetables and high intakes of unhealthy processed food. This may be a factor in the rising prevalence of obesity. A reason for the lack of diversity in children’s diets may be food neophobia. This study aimed to promote a healthy and varied diet among toddlers in kindergarten. The primary objectives were to reduce food neophobia in toddlers, and promote healthy feeding practices among kindergarten staff and parents. Secondary objectives were to increase food variety in toddlers’ diets and reduce future overweight and obesity in these children.

**Methods:**

This is an ongoing, cluster randomized trial. The intervention finished in 2014, but follow-up data collection is not yet complete. Eighteen randomly selected kindergartens located in two counties in Norway with enrolled children born in 2012 participated in the intervention. The kindergartens were matched into pairs based on background information, and randomly assigned to the intervention or control groups.

A 9-week multi-component intervention was implemented, with four main elements: 1) kindergarten staff implemented a pedagogical tool (Sapere method) in daily sessions to promote willingness to try new food; 2) kindergarten staff prepared and served the toddlers a cooked lunch from a menu corresponding to the pedagogical sessions; 3) kindergarten staff were encouraged to follow 10 meal principles on modeling, responsive feeding, repeated exposure, and enjoyable meals; and 4) parents were encouraged to read information and apply relevant feeding practices at home. The control group continued their usual practices. Preference taste tests were conducted to evaluate behavioral food neophobia, and children’s height and weight were measured. Parents and staff completed questionnaires before and after the intervention. Data have not yet been analyzed.

**Discussion:**

This study provides new knowledge about whether or not a Sapere-sensory education and healthy meal intervention targeting children, kindergarten staff, and parents will: reduce levels of food neophobia in toddlers; improve parental and kindergarten feeding practices; improve children’s dietary variety; and reduce childhood overweight and obesity.

**Trial registration:**

ISRCTN74823448 DOI 10.1186/ISRCTN74823448

## Background

A healthy diet is important for children’s development and growth during infancy and toddlerhood [1]. A healthy diet is also important for future health and morbidity, especially for non-communicable diseases such as diabetes, coronary heart diseases, and cancer [2]. There is concern about the composition of and lack of diversity in some children’s diets [3]. Some researchers suggest that a lack of diversity with a corresponding low intake of fruit and vegetables and high intake of unhealthy processed food, is caused by reluctance to try new food [4], and may be a contributing factor to the rising prevalence of obesity [3, 5, 6].

Taste preferences and food habits develop during infancy [7]. When solid food is introduced, the child is exposed to a diversity of foods and drinks, which may be the foundation for healthy and diverse eating habits [3]. Eating a variety of foods is essential to achieve adequate macro- and micronutrient intakes, and those with more varied food choices have better dietary quality [8]. Food neophobia, generally defined as reluctance to eat or avoidance of new foods, has become a central concept in discussions of diet and diversity, with food reluctance affecting both the quality of what children eat and what is served [3]. Food neophobia increases from around the age of 2 years, and decreases later in childhood [3]. An Australian study with 2- to 5-year-old preschoolers found that food neophobia was associated with lower intake of specific foods, such as vegetables [4]. They also found food neophobia was associated with children liking fewer types of food, having less variation in their food preferences, having a higher number of untested foods, and less healthy food preferences in general [4]. An American study aiming to increase preschoolers’ willingness to try new foods found that children started to like new food when they were exposed several times, and were given opportunity to explore, experience, and eat new food in a positive learning environment [9].

There are no studies in Norway that have specifically evaluated the lack of diversity in toddlers’ diets, or levels of food neophobia. Results from national dietary surveys of children aged 12 and 24 months showed that their diets were mostly consistent with current guidelines [10, 11]. However, about 17 % still ate “baby food” at age 24 months, and generally had a low intake of vegetables and high intake of processed food. Correspondingly, there is a high prevalence of overweight among infants and toddlers [12]. The most recent estimates from Norway show that 11–16 % of Norwegian children are overweight or obese before they reach the age of 6 years [12]. A possible way to reduce food neophobia and increase dietary diversity may be to increase levels of home cooking (and reduce processed food), thereby increasing the number and variety of foods presented to children [13]. This may potentially reduce the number of overweight children [6]. In a study conducted in the United Kingdom, vegetable consumption increased when 2- to 5-year-olds ate home cooked food [14]. However, there are no available studies focused on home cooked food.

Parents and other caregivers are central to a child’s food intake and meal patterns [15]. They decide what kind of food is bought and served, and are also involved through their food parenting style. Birch and colleagues argued that to provide guidance on responsive feeding, recognizing hunger and fullness and setting limits are important in interventions to improve children’s diets and prevent obesity [15]. There are several studies showing associations between parental food-related behaviors, feeding practices or food parenting, and children’s weight status [16]. However, most studies are cross sectional, and few have explored whether or not they can change parents/caregivers feeding practices [16].

Toddlers in Norway spend much of their time in kindergarten [17]; food and feeding practices in kindergartens influence children’s diet and eating habits, which in turn affects public health [18]. Mikkelsen et al. recently reviewed healthy eating interventions in preschools and concluded there was a scarcity of properly designed healthy eating interventions that used clear indicators and verifiable outcomes [19]. A national survey of meals in Norwegian kindergartens found that one of the most important challenges is the low number of kindergartens offering vegetables, with only 36 % serving vegetables every day [20]. This highlights a need for dietary interventions in kindergartens. The survey asked kindergarten staff what they thought were the most important factors for healthy food and meals in kindergarten; 49 % said staff attitudes were important, while 47 % thought that more courses were important [20]. This highlights the need for dietary interventions in kindergartens that include staff training and provision of pedagogical tools.

A possible solution is to educate kindergarten staff about the Sapere method, based on Puisais’ work [21]. This is a way of learning about food through senses (sight, touch, smell, taste, hearing) and language in kindergartens and schools. It aims to promote the building of healthy, balanced eating habits which help to prevent overweight [21]. At ages 2–3 years, children start to categorize and reason about foods [22]. Sensory lessons and meals based on the Sapere method aim to give children food experiences that help them recognize, differentiate, categorize, and reason about foods, and arouse curiosity about new foods [21]. Although the Sapere method is used in several European countries, there is limited research on its effect on child health [23]. In Norway, there is a lack of methods for use in kindergartens to introduce children to varied and balanced diets. To our knowledge, the Sapere method has not previously been tested in Norway; therefore, we used this method in the present intervention.

### Objectives and outcomes

The present study aimed to provide new knowledge about promoting a healthy and varied diet in toddlers by reducing levels of food neophobia. The intervention may also help to prevent future overweight. The intervention was multi-component intervention and used social ecological thinking, and included individual-level predictors as well as social and physical environmental determinants. The intervention was based on theories of healthy eating habits and food neophobia.

Our specific research question was: Can an intervention involving Sapere-sensory education and healthy meal preparation targeting children, kindergarten staff, and parents reduce levels of food neophobia in children, improve parental and kindergarten staff feeding practices, improve the variety and healthiness of children’s diets, and reduce future child obesity?

#### Primary outcomes

1. Children’s levels of food neophobia; assessed at baseline, after the intervention, and when the child reaches the age of 4 years.
2. Parental and kindergarten staff feeding practices; assessed at baseline and after the intervention, and parental feeding practices assessed when the child reaches 4 years.

#### Secondary outcomes

3. Child dietary habits and food variety; assessed at baseline, after the intervention, and when the child reaches 4 years.
4. Child body mass index and weight; assessed before and after the intervention, and when the child reaches 4 years.

#### Other study outcomes

Other study outcomes are: parental food intake, food variety, level of food neophobia, self-reported weight and height; and the level of food neophobia in kindergarten staff along with their self-reported cooking skills, knowledge, and attitudes relating to food.

## Methods

### Study design

This study used a cluster randomized controlled design to test a multi-component kindergarten-based intervention to improve dietary habits in toddlers. The study started in 2014 with follow-up studies planned for 2016 and 2017.

In total, 18 kindergartens participated in the study, with the director of each kindergarten consenting to participate on behalf of the kindergarten. Norwegian kindergartens are organized in different ways, but are usually divided into different departments. Two of the participating kindergartens had two departments for toddlers (aged 0–3 years), giving a total of 20 participating departments. These departments were randomized to either the control or intervention group.

The pedagogical leaders in the participating departments distributed a short invitation letter in both paper and electronic versions (an email link) to the parents, and a paper version to kindergarten staff. More detailed information about the study was provided on the study’s web page, and parents and kindergarten staff provided consent to participate via the web page.

The children’s parents completed a questionnaire covering food neophobia, parental feeding practices, food variety, and background variables at baseline and just after the intervention. Parents in the intervention group also answered evaluation questions after the intervention. Preference taste tests were conducted to assess the children’s behavioral food neophobia at baseline and after the intervention. Further follow-up is planned when the children are aged 4 years, in which parents will complete a short questionnaire with key questions. The children’s weight and height were measured pre- and post-intervention, and will be collected from health cards at age 4 years.

Participating kindergarten staff completed questionnaires before and after the intervention about food neophobia, feeding practices, cooking skills, and food-related knowledge and attitudes. Staff in the intervention group also answered evaluation questions after the intervention. In addition, pedagogical leaders were asked to assess the implementation of the intervention.

The intervention occurred over a 3-month period. The intervention program ran 3 days per week for 9 weeks; with 3 weeks without intervention topics (see detailed description below). The control group continued with their usual practices.

The baseline study for this randomized control trial using child and parent data drew on a larger cross-sectional sub study entitled “Preschoolers’ food courage,” in which all kindergartens in the counties of Vest-Agder and Aust-Agder, Norway, were invited to participate. In total, 266 kindergartens participated and 510 parents completed questionnaires. For parents who participated in the present intervention but not in the cross-sectional study, and had therefore not completed the baseline questionnaire, the baseline questionnaire was completed before the intervention (*n =* 88).

### Study population

The primary study population was 2-year-olds. Inclusion criteria for children were: born in 2012, attended the included kindergartens in Vest-Agder and Aust-Agder, were in kindergarten departments for toddlers, and had a parent that understood Norwegian.

There were 183 children enrolled in the 18 kindergartens who met the inclusion criteria. Of these, 116 children participated (participation rate, 63 %). There were 69 children in the intervention group and 47 children in the control group.

Of the participating children, 104 participated in preference taste test 1 (baseline) and 101 in preference taste test 2 (after intervention). There were 90 parents who completed the baseline questionnaire and 87 who completed the second questionnaire.

There were 89 kindergarten staff (assistants and pedagogical leaders); 82 participated at baseline (participation rate, 92 %). In total, 43 kindergarten staff participated in the intervention group and 39 in the control group. In the post-intervention study, 78 completed the questionnaire. There were 18 directors in the participating kindergartens, all of whom completed a questionnaire collecting descriptive information about the kindergartens and food offered in that kindergarten.

### Sample size calculation

Sample size was originally calculated according to the secondary weight outcome. For this outcome we wanted to observe a difference of 1 kg in weight over time between the two groups. According to a national survey, the standard deviation (SD) of 2-year-olds’ weight is 1.6 [11], with a power of 80 % and significance level of 5 %; therefore, 41 children needed to be recruited for each group. Due to possible dropout over time, we increased this to 45 children in each group. At the start of the study, we did not have a relevant SD for our primary outcome (level of food neophobia) in this age group. Our previous cross sectional trial of 510 2-year-olds (not yet published) indicated that the SD for food neophobia was 9.4, and a mean score reduction in the level of food neophobia from 18.2 to 12.0 would be of public health value. Using a power of 80 % and significance level of 5 %, this suggested 37 participants were needed in each group. Therefore, we aimed to include 10 kindergartens each in the control and intervention groups, and recruit about 45 children for each group.

### Randomization

Of the 266 kindergartens in the previous cross-sectional study, 144 had eight or more toddlers born in 2012 who were eligible for inclusion in the present cluster randomized trial. Of these, 50 kindergartens in the two Agder counties were randomly selected and invited to participate in the cluster randomized trial. After contacting the selected kindergartens, we realized that not all kindergartens had all enrolled 2012 children in one department. Therefore, we changed the kindergarten inclusion criterion from eight to a minimum of seven toddlers born in 2012 in one department. There were 42 kindergartens that satisfied this new inclusion criterion. Of these, 18 kindergartens agreed to participate and were matched into pairs. Kindergartens were matched according to urbanity, number of 2012-born children enrolled, and whether they had previously focused on nutrition. The pairs were then randomly allocated to the intervention or control group, with nine kindergartens (10 departments for toddlers) in each group. The kindergartens were informed about their group allocation after agreeing to participate. In the intervention group, one kindergarten withdrew from participation due to closing of the kindergarten, and one kindergarten in the control group had no parents of children who agreed to participate, leaving only the baseline results questionnaires from staff. In total, 16 kindergartens with 18 departments for toddlers participated in this study.

### Intervention

The multi-component intervention targeted children and kindergarten staff, with some parts of the intervention also targeting parents. The intervention period lasted for 3 months, divided into three periods of 3 weeks of active involvement, and two breaks of 1 and 2 weeks respectively. These breaks were due to kindergarten holidays. Kindergarten staff in the intervention group attended a class at the university conducted by one of the present authors (SHH) to learn about the Sapere method and intervention elements. All staff were given a handbook entitled “Overall plan for the intervention” to guide them in implementing the program. This included a timetable, 10 meal principles, and a template for the Sapere-sensory lessons. They also learned food preparation and time-saving tips. The pedagogical leaders in the intervention group also attended a class to learn about food neophobia, development of healthy eating habits early in life, and how kindergartens can play a role in healthy eating in children.

Thematically, there were four main intervention elements involving the kindergartens. The first element was implementation of Sapere-sensory education in the kindergartens’ pedagogical group sessions. During daily lessons, children were introduced to “the week’s vegetable”; each vegetable was presented three different ways on the 3 intervention days in that week. The first day it was presented raw, the second day raw with dip, and on the third day it was presented differently (e.g., baked, mashed, pickled). The kindergarten staff in the intervention group were asked to follow a template when conducting these sessions.

Children were divided into groups (maximum of six in each group). Each session started with a soft toy dog visiting the children. The “dog” was the presenter of the week’s vegetable, which was placed in a large pink box. After the box was opened and the vegetable revealed, staff showed the children cards with photos of our five senses (touch, sight, smell, taste, and hearing), and encouraged the children to point at their own senses and pronounce the names of senses and foods. Children investigated the vegetables further through five questions: 1) What does this vegetable look like?; 2) How does it feel to the touch?; 3) Is there any smell?; 4) What does it taste like?; 5) Are there sounds while chewing? As toddlers need repetition to learn concepts, the same five questions were asked each intervention day. Staff helped with descriptions as toddlers’ have a limited vocabulary. Some words were written down and repeated in the next session. Sheets with the words and photos from the sessions were kept in a binder. The template described above was developed in cooperation with staff at Gunghästen kindergarten in Sweden, which has several years of experience with the Sapere method. To get started, the kindergarten received 2–3 Sapere kit boxes (a large box including the soft toy dog, cards, kitchen materials) and a book about senses and food [24]. In total, nine vegetables were presented using the Sapere method over the nine weeks (Table 1).

**Table 1:** The week’s vegetables used in Sapere-sensory education

Week	Vegetable
1	Cucumber
2	Broccoli
3	Fennel
4	Radish
5	Beets
6	Spinach
7	Cauliflower
8	French beans
9	Butternut squash

All children in the participating departments attended the Sapere sessions, including non-participating children.

The second intervention theme was that children were offered a cooked, healthy lunch prepared at the kindergarten on the intervention days over the 9-week period. All children in the department were offered the same food, including non-participants. In Norway, it is not common to have a chef in the kindergarten [20]. Detailed descriptions on how to cook the nine selected dishes were provided to kindergarten staff (Table 2). The children were not included in the food preparation. Several dishes were prepared and tested before the study started by a skilled cook (one of the present authors, SHH) to determine the included dishes. These dishes were intended to give the children experience with novel healthy foods with varied color, texture, odor, sound, and the five basic tastes. The selected dishes were organized into three blocks: each block consisted of a fish dish, a meat dish, and a vegetarian dish (Table 2). Children were exposed to each block three times before they were exposed to the next block of dishes. Through lunches and Sapere sessions, children were exposed a minimum of six times to each vegetable listed in Table 1. The menu was based on recipes from *Food enjoyment,* a cookbook by Claus Meyer [25]. The Danish Health Department had calculated the nutrient content of each dish. Kindergartens in the intervention group were assigned equipment to make cooking easy and feasible. They also received three different handbooks developed for the project “Home cooked food guide,” and three food kit boxes (including spices, herbs, vinegar, mustard, and so forth); one for each time period. Financial compensation for food costs was provided after the intervention period.

**Table 2:** Lunch dishes cooked in the intervention kindergartens

	Meat	Fish	Vegetarian
Block 1	Chicken skewers with barley and pea puree	Fish cakes with vegetable remoulade	Omelet with potato
Block 2	Homemade chicken salad	Fish lasagna	Potato and leek soup with yogurt
Block 3	Lamb meatballs with wheat bulgur salad	Roasted fish fillet with carrot puree	Butternut soup with orange and crunchy bread

The third intervention theme was that kindergarten staff integrated 10 meal principles about feeding practices (Table 3). These principles were explained during the sessions and were intended to improve kindergarten feeding practices. The principles supported serving the selected healthy dishes, encouraging staff to be good role models, promoting responsive feeding practices, and contributing to a positive food environment for the children.

**Table 3:** The 10 meal principles for the intervention kindergartens

	Meal principle
1	Eat together with the children in your group, and contribute to a relaxed and enjoyable meal
2	Be aware that you are a role model at the table
3	Adults are the ones who decide the kind of food that is served
4	Always serve the lunch dishes with a positive attitude
5	Respect children’s right to have their own tastes; no taste is right or wrong
6	Encourage the children to serve themselves, providing guidance, assistance, and support
7	Encourage the children to eat with the least assistance
8	The child decides if he/she will eat or not. They are allowed to be picky
9	Be aware and respect the child’s signals of fullness, thirst, or hunger
10	Never use food or beverages as rewards, punishments, or emotion regulation

The fourth element was involving parents to improve their feeding practices. Parents were given short postcard messages corresponding to the 10 meal principles. There was also dialogue with the parents through posters in the kindergarten’s wardrobe about the current week’s menu, vegetable (picture), and the current day’s dish (pictures of ingredients). The kindergartens received preprinted postcards and posters for each 3-week period.

The control group continued with their usual pedagogical sessions, meals, and food serving practices and the parents did not receive any information.

### Measurement instruments

#### Measures of child food neophobia

Child food neophobia was measured with a questionnaire and a preference taste test. Parents completed a questionnaire which included a 6-item version of Pliner’s 10-item Food Neophobia Scale (FNS) [26]. This version is commonly used; for example, in British samples [27]. We performed a preference taste test, which tested the child’s willingness to try known and unknown foods, also based on Pliner’s work [26]. A pilot with toddlers from a non-participating kindergarten was conducted before the study started. Some minor adjustments on how the preference taste test was conducted were made before it was administered at baseline and repeated 3–5 weeks after the intervention period.

The preference taste test was conducted in the kindergartens. Participating children in the intervention and control groups were taken out of ordinary play and were asked to sit at a table and informed about the procedure. Each child was presented four supposedly known foods, and then four supposedly unknown foods. In addition to these eight foods, there were two familiar, well-liked foods. For practical reasons, all food was served cold. The children were presented these foods sitting with 2–5 of their peers. The foods were organized into two blocks; two novel and two familiar foods, and one familiar well-liked food in each block. First, the children were offered a familiar well-liked food, then every second time were offered a familiar and a novel food (Table 4). They were presented the foods one at a time and were told the name of the food. Each child was asked in a friendly way “Do you want to taste this? You can say yes or no.” Children who were willing to try, got a small sample on their plate and were asked not to taste before all foods in that block were introduced. They were then told that they could taste the selected foods if they wanted. Two project managers were present and recorded on a precoded sheet how many food items the child selected, and how many food items the child actually tasted. They also recorded whether or not the child spat out the food. The food was presented in a standardized way. Effort was made to put the children at ease by having a teddy bear a present the foods and known kindergarten staff always present.

**Table 4:** Foods presented at the preference taste tests, according to familiarity

		Baseline		After the intervention	
Order of introduction	Familiar/novel foods	Block 1	Block 2	Block 1	Block 2
1	Familiar well liked foods	Sweet biscuits	Banana	Sweet biscuits	Ritz cracker
2	Familiar foods	Cauliflower	Peas	Cultured milk, strawberry flavor	Raspberry
3	Novel foods	Kidney beans	Butternut squash	Blackcurrant	Cultured milk, natural
4	Familiar foods	Whole wheat bread	Grilled chicken	Baked salmon	Sautéed onion
5	Novel foods	Roasted lamb	Rye bread	Brussels sprouts	Salt herring

#### Measures of parental and kindergarten staff feeding practices

Parental and kindergarten staff feeding practices were measured with the Comprehensive Feeding Practices Questionnaire (CFPQ), which is age appropriate and has been validated [28]. Kindergarten staff completed a moderated version of the CFPQ, adapted to a kindergarten context.

#### Measures of children’s dietary habits and food variety

Child food intake and diet variety were measured by Food Frequency Questionnaire (FFQ), 43 questions administered before and after the intervention. These included questions on how often the child ate: fruits, berries, vegetables, potatoes, rice, pasta, bread, cereals, porridge, unprocessed meat and fish, processed foods, snacks, and questions about beverage intake. There were 10 response options ranging from “never” to “several times a day.”

#### Measures of weight and height

During the visits to the kindergartens for the preference taste tests, the children’s height and weight were measured. Height was measured with a portable stadiometer (Seca 217) and weight with a digital scale (Seca 877) at baseline and after the intervention, and will be self-reported at age 4 years. The digital scale was able to weigh two persons at a time, and this was used on some occasions when a child did not want to be weighed alone. Body mass index was calculated according to the Extended International Body Mass Index Cut-Offs for Thinness, Overweight and Obesity in Children [29].

#### Measures of other variables

Parental food intake was measured with FFQ-questions, and food neophobia with a 6-item version of the FNS. Questions about length of education, breastfeeding, introduction to solids, and meal patterns were also included. After the intervention, evaluation questions were included for parents in the intervention group.

Food neophobia in kindergarten staff was measured with the same 6-item FNS. Staff cooking skills were measured with a newly developed and validated cooking skill scale by Hartmann et al. [30]. After the intervention, evaluation questions were included for staff in the intervention group.

### Compliance with intervention elements

The pedagogical leaders in the intervention group completed evaluation sheets every day during the intervention weeks. They were asked to assess the implementation of the intervention elements on a scale of 0–10.

### Statistical analysis

#### Descriptive statistics

Data have not yet been analyzed, as some data collection is still to be performed. Normally distributed quantitative data will be analyzed using means and SD. Data that are not normally distributed will be reported with medians and interquartile ranges.

#### Multivariate analysis

Our primary goal is to detect differences in food neophobia scores between the intervention and control groups. As the study design involved pre- and post-test measures and clustering, data will be analyzed with linear mixed models. This method will also be used to analyze the secondary outcome measures.

## Discussion

There has been a call for intervention studies in preschools to promote healthy diet and weight development [19]. Recent studies have raised concern about the lack of variety in children’s diets, and have related this to the rise of overweight and obesity in preschool children [3]. With the study “Preschoolers’ food courage” we are investigating the effectiveness of a multi-component kindergarten-based intervention to: reduce food neophobia; increase food variety; increase healthy feeding practices in kindergarten and at home; and over time, reduce the prevalence of overweight in this group.

We developed an intervention that may be easily implemented in kindergartens. The intervention kit includes four elements: a pedagogical tool (Sapere method), menu of associated lunch dishes, 10 practical tips for healthy eating in children, and postcard messages for parents.

The strengths of our study are that it is being conducted in a natural setting, making it possible to reproduce in other kindergartens if it shows an effect. Further, the use of the Sapere method, which was developed for children, makes it relevant for the kindergarten setting. The Sapere method is widely used in some countries; however, few studies have evaluated its effect on children’s diet and health [21]. Methodological strengths include objective measurements of children’s willingness to try new foods, and children’s weight and height at baseline and after the intervention.

However, our study also has some limitations. Recruitment of kindergartens and parents was difficult, and although we included the desired number of children, we should have included more children to have full datasets for 37 children in each group, as suggested by the power calculations; especially as a somewhat higher number may be needed due to the clustering effects. In the preference taste tests, we found that not all children were present on the day we arrived, and although we returned to some kindergartens for baseline measures, it was difficult to test all participating children. Further, it may be difficult to track the children at age 4 years, when they change kindergarten departments at age 3 years. Another limitation is generalizability, as only children from the two southern counties in Norway were included.

## Conclusion

The study results will provide new knowledge on whether or not Sapere-sensory education and a healthy meal intervention targeting children, kindergarten staff, and parents will: improve variety in children’s diets, reduce food neophobia, improve parental and kindergarten feeding practices, and reduce childhood overweight and obesity.

### Ethics approval and consent to participate

The protocol for the present study was notified to the Norwegian Social Science Data Services, Data Protection Official for Research, 26/03/2014, reference 37459. Informed consent was obtained from parents of all participating children and from all participating kindergartens and kindergarten staff.

### Consent for publication

Not applicable.

### Availability of data and material

We do not wish to share our data before we have thoroughly analyzed it.

## Abbreviations

CFPQ: comprehensive feeding practices questionnaire
FNS: food neophobia scale
FFQ: food frequency questionnaire
